# Synergistic Effects of 1-Octyl-3-Methylimidazolium Hexafluorophosphate and Cellulose Nanocrystals on Improving Polyacrylate Waterborne Anti-Corrosion Coatings

**DOI:** 10.3390/polym15040810

**Published:** 2023-02-06

**Authors:** Zeping Wang, Binjie Hu, Haibin Yu, George Zheng Chen

**Affiliations:** 1 Key Laboratory of Carbonaceous Wastes Processing and Process Intensification of Zhejiang Province, Department of Chemical & Environmental Engineering, University of Nottingham Ningbo China, 199 Taikang East Road, Ningbo 315000, China; 2 Ningbo Institute of Materials Technology and Engineering, Chinese Academy of Sciences, Ningbo 315201, China; 3 Department of Chemical and Environmental Engineering, Advanced Materials Research Group, Faculty of Engineering, University of Nottingham, Nottingham NG7 2RD, UK

**Keywords:** ionic liquid, cellulose nanocrystals, corrosion protection, polymer, electrochemical analysis, composite

## Abstract

In this study, three copolymers of poly(methyl methacrylate) and poly(butyl acrylate) (PMMA-co-PBA) latex containing 1-octyl-3 methylimidazolium hexafluorophosphate (C_8_mimPF_6_), cellulose nanocrystals (CNCs), and C_8_mimPF_6_-CNCs were successfully synthesized through mini emulsion polymerization. These novel composites were each coated on mild steel panels and tested for their anti-corrosion performance by immersion of the coated samples in 3.5 wt% sodium chloride (NaCl) solution over a certain period. The synergistic anti-corrosion effects of the C_8_mimPF_6_-CNCs sample led to the highest coating resistance, charge transfer resistance, and corrosion inhibition efficiency and the lowest diffusion coefficient and corrosion rate. The proposed synergistic mechanism revealed that CNCs enhanced the barrier effect of the coating while C_8_mimPF_6_ inhibited corrosion when released.

## 1. Introduction

The ionic liquid C_8_mimPF_6_ as a corrosion inhibitor has been proven to be effective in waterborne anti-corrosion coatings on mild steel in simulated seawater, i.e., 3.5 wt% NaCl solution [1]. The mechanism of C_8_mimPF_6_ in corrosion inhibition is mainly ascribed to the adsorption of imidazolium ions to passivate the reaction sites on the metal surface, which in turn prevents corrosion from occurring when encapsulated C_8_mimPF_6_ is released into the corrosive medium [1,2]. Although the anti-corrosion performance was enhanced with the introduction of C_8_mimPF_6_, it was still inferior to other anti-corrosion coatings, such as epoxy, due to the poor performance of neat waterborne PMMA-co-PBA [3]. However, as one of the acrylate coatings, waterborne PMMA-co-PBA coatings have advantages such as low cost, excellent adhesion, coalescence, color retention, and satisfactory UV and hydrolysis resistance [4,5]. Rather than searching for alternative waterborne coating resins without prior anti-corrosion resistance to encapsulate C_8_mimPF_6_, direct addition of fillers to enhance the barrier effect of the neat PMMA-co-PBA coating is a facile route to improve the anti-corrosion performance [6]. 

Cellulose, a natural filler, is fibrous, tough, water-insoluble, biodegradable, biocompatible, renewable polymer, and is also abundant in nature [7]. Cellulose can form nanocrystals (CNCs) which, unlike cellulose nanofibers (CNFs) with highly entangled web-like structures, present shorter lengths and needle-like shapes [8]. Hydroxyl groups in CNCs lead to strong hydrogen bonds and a highly crystalline structure [7]. Moreover, CNCs can act as a gas or water-impermeable barrier and additives can enhance the mechanical properties of coatings [9,10]. He et al. [11] incorporated CNCs into a waterborne acrylate coating and compared the anti-corrosion performance before and after CNC addition. They found that the polarization resistance of the coating with CNCs slightly increased after 35 days of exposure, whilst the water uptake was relatively stable at a low level. On the contrary, the polar resistance of the coating without CNCs decreased after 21 days of exposure, with the coating showing remarkable degradation. They ascribed the enhancement effect to hydrogen bonding between CNCs and other coating materials. Therefore, we are interested to introduce CNCs as fillers into our previously reported ionic liquid-containing anti-corrosion coating formula [1]. Previous reports have shown that iridescent PVA composite films can be fabricated with the combination of CNCs and ionic liquids [12]. This composite is of high strength and toughness [13] and can adsorb and recover lithium ions from groundwater [14]. These works reveal that CNCs and ionic liquid could be homogenized in one functioning composite. So far, no research work has reported such an idea to combine C_8_mimPF_6_ and CNCs into a waterborne acrylate coating. To the best of our knowledge, we are the first to incorporate C_8_mimPF_6_ and CNCs as a corrosion inhibitor and barrier into coating fillers. In this research, systematic characterizations and analyses on the anti-corrosion performance were carried out, exploring their synergistic effect and the underlying anti-corrosion mechanism. 

## 2. Materials and Experimental Methods 

### 2.1. Materials

Methyl methacrylate (MMA, CP), sodium dodecyl sulfonate (SDSO, CP), L-Ascorbic acid (AAc, AR), and hydrogen peroxide (H_2_O_2_, AR) were purchased from Sinopharm Chemical Reagent Co., Ltd, Shanghai, China. Hexadecane (HD, 98%) and n-butyl acrylate (BA, 99%) were purchased from Aladdin Industrial Inc, Shanghai, China. Cellulose nanocrystals (CNCs) were kindly supplied by ScienceK Co., Ltd, Huzhou, China. The compound 1-octyl-3 methylimidazolium hexafluorophosphate (C_8_mimPF_6_, 99%) was purchased from Cheng Jie Chemical Co., Ltd, Shanghai, China. Ultra-pure water with a resistivity of less than 18.2 MΩ cm was used in all experiments. A mild steel panel (steel Q235) for testing was bought from Zhi Bao Metal Products Co., Ltd, Shenzhen, China. The mild steel composition (wt%) was 0.14–0.22 C, 0.3–0.65 Mn, < 0.30 Si, < 0.045 *p*, < 0.055 S, and the remaining was Fe.

### 2.2. Preparation of Mini Emulsion

The mini emulsion was prepared by mixing the oil phase containing a 1:1 mass ratio of MMA to BA. C_8_mimPF_6_ (10 wt%) and 0.5 wt% CNC were added to the oil and water phase, respectively, before phase mixing. A sonicator (Scientz II, Xinzhi Co., Ltd, Ningbo, China) was used to homogenize the two-phase mixture using a power of 285 W for 6 min with a pattern of 1 s on and 1 s off.

### 2.3. Mini Emulsion Polymerization

Polymerization was carried out in a 250 mL four-necked flask equipped with a stirrer, a reflux condenser, a thermometer, and a nitrogen inlet. The flask was immersed in a water bath with the temperature controlled at 40 ℃. The mini emulsion was loaded into the flask and stored under stirring and nitrogen bubbling for 1.5 h to remove oxygen. After the reaction temperature (40 ℃) was reached, a 0.1 mol% H_2_O_2_/AAc solution with a molar ratio of 1 to 1.3 was injected to start the reaction.

### 2.4. Coating Film Characterization

#### 2.4.1. Testing Sample Preparation

Before characterization, the copolymer coating film was prepared as follows. After treatment with emery paper, mild steel specimens of 1 cm in length and 1 cm in width were selected as the substrate for coating. After treatment with emery paper, several drops of latex developed in previous steps were dropped onto one side of the surface of the mild steel specimen, followed by the rolling of bar coater BGD212/150μm from one side to the other side evenly. After evaporation of water, the solid content remained. The thickness of the film was measured with a PosiTectorFS1 apparatus, and the film thickness was controlled at 40 ± 2 µm. 

#### 2.4.2. FTIR

FTIR spectra of copolymer coating samples were collected using Vertex 70, Bruker Co., Ltd, Beilin, Germany. The operation was carried out over the wavenumber range of 4000 to 400 cm^−1^ with a resolution of 4 cm^−1^. 

#### 2.4.3. Surface Morphology

The surface morphology of the coating film was investigated by a scanning electron microscope (SEM) (ΣIGMA/VP, Carl Zeiss Microscopy Ltd., Jena, Germany) at an accelerating voltage of 4 kV.

#### 2.4.4. Wettability

The contact angle between water and the coated mild steel surface was measured with a dynamic contact angle measuring instrument (Theta Flex, Biolin Scientific Co Ltd., Shanghai, China). Initially, 5 μL of water droplets was loaded on the tip of a needle tube, and after dropping onto the coated mild steel surface, the contact angle was recorded at the 20th second. The contact angle measurements were repeated three times for each sample. An average value was used for the final measurement result. 

#### 2.4.5. Electrochemical Techniques

The anti-corrosion performances of the coatings were measured using Princeton’s electrochemical workstation. A conventional three-electrode configuration electrochemical cell was prepared with a volume of 30 mL. The mild steel electrode, a platinum sheet, an Ag/AgCl electrode (in 3.0 M KCl), and 3.5 wt% NaCl (aqueous) were used as the working, counter, and reference electrodes and the electrolyte, respectively. The open circuit potential (OCP) for each sample was measured for 0.5 h before electrochemical impedance spectroscopy (EIS) and potentiodynamic polarization curves were measured. The EIS test was performed at the OCP with a frequency range of 10 kHz to 10 mHz under ±10 mV amplitude sinusoidal voltage. Tafel plots were scanned at rate of 0.5 mV/s in the range of ±250 mV VS.OCP. 

## 3. Results and Discussion

The latex was prepared as described in Section 2.3. The anti-corrosion performances of the copolymer coatings containing C_8_mimPF_6_ and CNCs were evaluated via open circuit potential (OCP), electrochemical impedance spectroscopy (EIS), and Tafel polarization curves. The status of C_8_mimPF_6_ and CNCs was revealed, and the mechanism of the synergistic effect was further explained in the following sections.

### 3.1. Anti-Corrosion Performance Evaluation of Copolymer Coating

#### 3.1.1. Open Circuit Potential (OCP)

**Figure 1:**
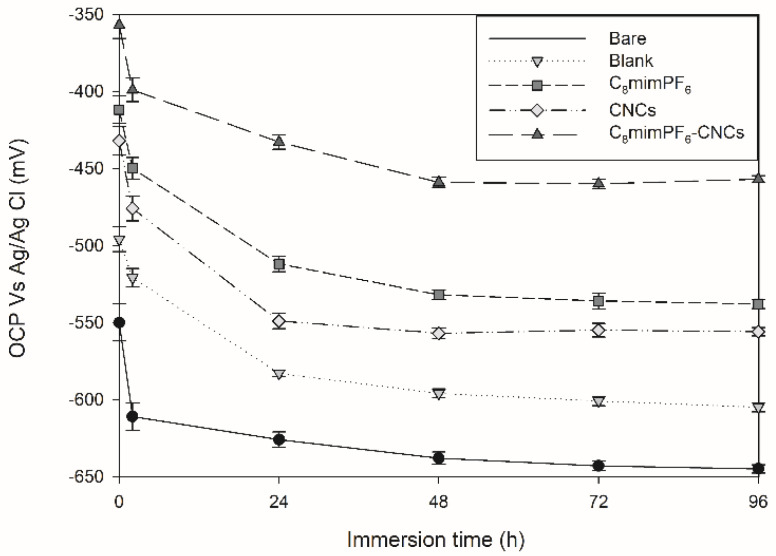
OCP for various samples at different immersion times.

The electrochemical technique started with open circuit potential (OCP) measurement. OCP indicates the thermodynamic tendency of a material under electrochemical oxidation in a corrosive medium [15]. In other words, OCP values can reflect the extent of corrosion. Severe corrosion exhibits a more negative OCP. The variation in OCP versus immersion time in the absence and presence of C_8_mimPF_6_ and CNCs is displayed in Figure 1. At the initial immersion stage, the OCP of bare (no coating layer), blank (with a neat PMMA-co-PBA coating), C_8_mimPF_6_ (PMMA-co-PBA coating with 10 wt% C_8_mimPF_6_), CNCs (PMMA-co-PBA coating with 0.5 wt% CNCs), and C_8_mimPF_6_^−^CNCs (PMMA-co-PBA coating with 10 wt% C_8_mimPF_6_ and 0.5 wt% CNCs) samples were approximately −550, −496, −412, −432, and −357 mV, respectively. The drop in OCP in the first 2 h was ascribed to the penetration of corrosive ions such as OH^−^ and Cl^−^ and oxygen through coating micropores and the coating/steel interface [16]. After 96 h of immersion, the OCP of bare mild steel reduced sharply from −550 mV to −645 mV, which indicated that bare mild steel was severely corroded. In addition, the OCP of blank, C_8_mimPF_6_, CNCs, and C_8_mimPF_6_-CNCs samples at 96 h were −605, −538, −556, and −457 mV, respectively. The steady OCP values of these samples from 48 h to 96 h was attributed to the saturated water absorption of the coating and the accumulation of corrosion products [17]. Among them, the lowest rate of declination was observed in the C_8_mimPF_6_-CNCs sample. Thus, it could be concluded that the C_8_mimPF_6_-CNCs sample exhibited the most pronounced anti-corrosive effect. 

#### 3.1.2. Electrochemical Impedance Spectroscopy (EIS)

After the stabilization of OCP, other electrochemical techniques such as EIS and Tafel were applied to further quantitively evaluate the extent of corrosion. For EIS, the resistance and capacitance of the coating could be directly obtained through data fitting. Moreover, with the obtained coating capacitance values, other indirect parameters such as the water absorption ratio of the coating and the diffusion coefficient of the corrosive ions could be calculated via the Brasher and Kingsbury equation [18] and the simplified Fick’s law of diffusion [3], respectively. 

**Figure 2:**
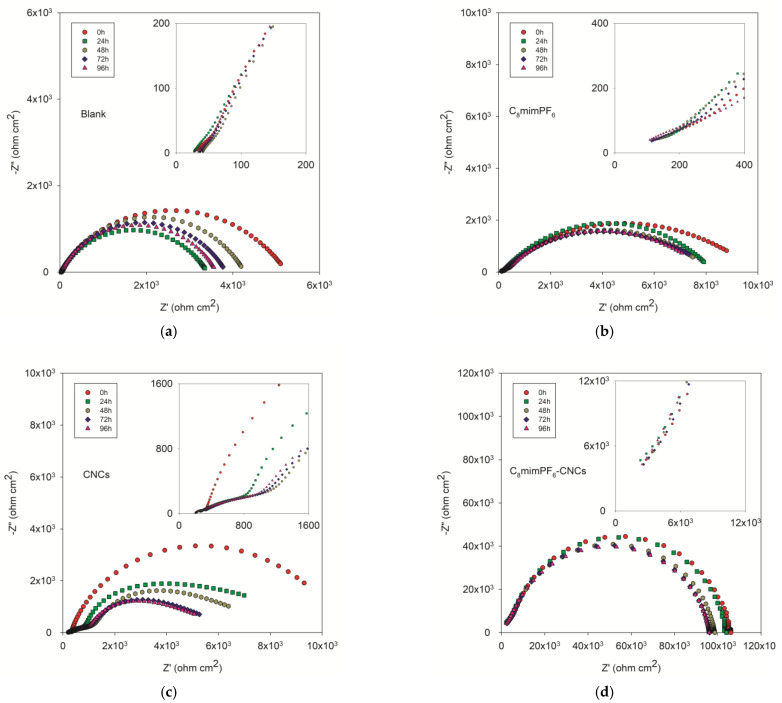
Nyquist impedance plots of coated samples after 96 h of immersion in 3.5 wt% NaCl solution. (**a**) Blank (neat PMMA-co-PBA coating), (**b**) C_8_mimPF_6_ (PMMA-co-PBA coating with 10 wt% C_8_mimPF_6_), (**c**) CNCs (PMMA-co-PBA coating with 0.5 wt% CNCs), and (**d**) C_8_mimPF_6_-CNCs (PMMA-co-PBA coating with 10 wt% C_8_mimPF_6_ and 0.5 wt% CNCs).

**Figure 3:**
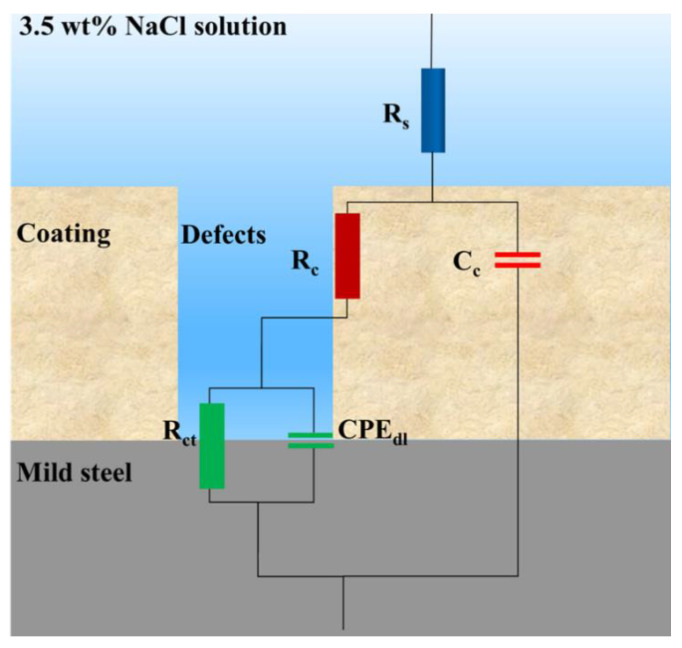
Equivalent circuit for EIS data fitting.

**Figure 4:**
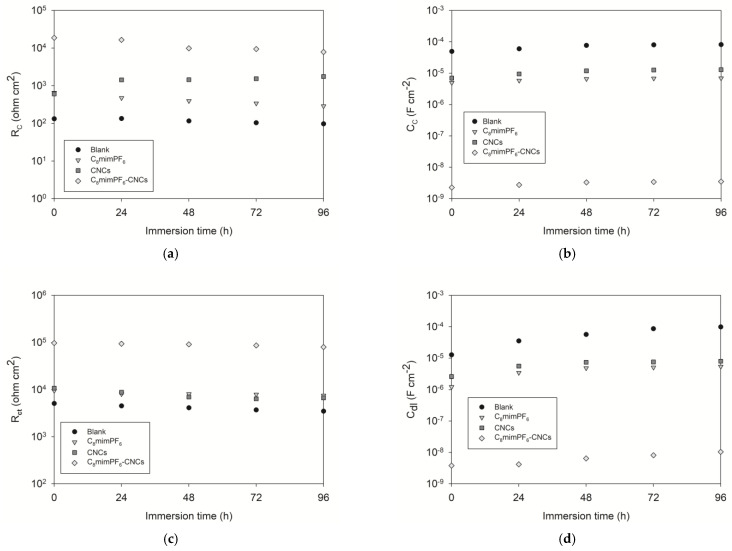
EIS parameters of coated samples in 3.5 wt% NaCl solution at different immersion periods. (**a**) Coating resistance, *R_C_*, (**b**) coating capacitance, *C_C_*, (**c**) charge transfer resistance, *R_ct_*, and (**d**) double-layer capacitance, *CPE_dl_*.

Figure 2 shows the Nyquist impedance plots of different coated samples after 96 h of immersion in 3.5 wt% NaCl solution. The Nyquist plot is one of the manifestations of EIS tests, with different parts representing different hierarchies of the coating. For instance, in Figure 2a, the bottom left part at high frequency was correlated to defects and pinholes of the coating, while the upper right part at low frequency was assigned to the interface of the coating and the mild steel surface [19,20]. The shrinking semicircles during the immersion test exhibit the declining anti-corrosion performance. The initial linear portion of the curve shown in Figure 2c could be attributed to the pseudo-two-time constant phenomenon caused by the penetration of electrolytes through the coating defects [21,22]. Moreover, an electrical equivalent circuit was applied to model Nyquist plots for quantitative evaluation. As shown in Figure 3, the circuits consisted of different electrochemical parameters such as solution resistance (*R_S_*), coating resistance (*R_C_*), coating capacitance (*C_C_*), charge transfer resistance (*R_ct_*), and constant phase element (*CPE_dl_*), where *CPE_dl_* represents the non-ideal capacitance of double layer. Here, CPE is a valid model to simulate the dielectric response, which symbolizes a real-life capacitive impedance independent of frequency [23]. Using ZSimpWin 3.60 software, the results of resistance and capacitance were fitted and presented in Figure 4. Figure 4a and c show the coating resistance (*R_C_*) and charge transfer resistance (*R_ct_*), respectively. 

*R_C_* indicates the barrier performance to ionic paths through the coating [24]. Generally, *R_C_* values decreased with immersion time, which reveals a degradation of the coating and a decline in anti-corrosion performance [25]. As Figure 4 shows, the *R_C_* value of the blank sample decreased from 132 Ω cm^2^ at the initial stage to 97 Ω cm^2^ after 96 h of immersion. Meanwhile, the *R_C_* of the C_8_mimPF_6_ sample reduced from 624 Ω cm^2^ to 142 Ω cm^2^. The reduction in coating resistance shows the degradation of the coating. The final *R_C_* for the C_8_mimPF_6_ sample was still higher than the blank sample, which indicates the prior anti-corrosion performance of the C_8_mimPF_6_ sample compared with the blank one. In contrast to the C_8_mimPF_6_ sample, the *R_C_* of the CNCs sample increased significantly from 603 Ω cm^2^ at the initial stage to 1754 Ω cm^2^ after 96 h immersion. One explanation for this is the enhancement of the barrier effect after introducing CNCs into the coating [10]. Another possible reason may be a pseudo-two-time constant phenomenon after the penetration of electrolytes [21,22], which interferes with the curve fitting, resulting in a larger R_C_ value than the real value. During the immersion process, the *R_C_* of the C_8_mimPF_6_-CNCs sample had the highest values of 18,670 and 7865 Ω cm^2^ at the initial stage and after 96 h of immersion, respectively. This value was almost 100 times higher than the *R_C_* of the blank sample, indicating the remarkable protective capability against corrosion with the combination of C_8_mimPF_6_ and CNCs. In addition, the charge transfer resistance, *R_ct_*, the resistance to charge transfer on mild steel, was inversely related to the corrosion rate [26]. Similar to the results of *R_C_*, the blank sample presented the lowest *R_ct_* at 3490 Ω cm^2^ after 96 h of immersion. The *R_ct_*s of the C_8_mimPF_6_ and CNCs samples were 7518 and 6677 Ω cm^2^, respectively, after 96 h of immersion, which was much higher than the *R_ct_* of the blank sample, indicating an enhancement in charge transfer resistance with effect of adding these two components. Surprisingly, the C_8_mimPF_6_-CNCs sample exhibited the highest *R_ct_* of 79,460 Ω cm^2^ after 96 h of immersion, which was nearly 22 times larger than the *R_ct_* of the blank sample and one magnitude larger than that of the C_8_mimPF_6_ or CNCs samples. After immersion tests, Lewis et al. [27] studied the corrosion resistance of a 60 µm-thick waterborne acrylic coating modified with nano-sized titanium dioxide and found that the highest resistance after 48 h of immersion in 3% (m/v) NaCl solution was approximately 18,000 Ω cm^2^. Our C_8_mimPF_6_-CNCs sample with a lower thickness and higher resistance is clearly more competitive, demonstrating that the lowest corrosion rate could be achieved under the synergistic effects of C_8_mimPF_6_ and CNCs. 

In addition to resistance, the coating capacitance (*C_C_*) and double-layer capacitance (*CPE_dl_*) also indicate the extent of corrosion; the increase in capacitance indicates a rise in electrolyte uptake [25]. This study revealed the synergistic effect of C_8_mimPF_6_ and CNCs on capacitance, presented as the electrolyte uptake (water absorption) ratio and diffusion coefficient of corrosive ions. The main component of the electrolyte is water; therefore, the electrolyte uptake in this experiment is mainly water absorption. The Brasher and Kingsbury equation [18] was applied to calculate the volume fraction of water absorption, ϕ %:

(1) $$\phi \%=\frac{log\left(\frac{C_{t}}{C_{0}}\right)}{log\varepsilon_{w}}\times 100\%$$

where *C_0_* and *C_t_* are the coating capacitance at the initial stage and time *t*, respectively. *ε_w_* is the dielectric constant of the electrolyte (78.3 at 25 °C for water [28]). 

**Figure 5:**
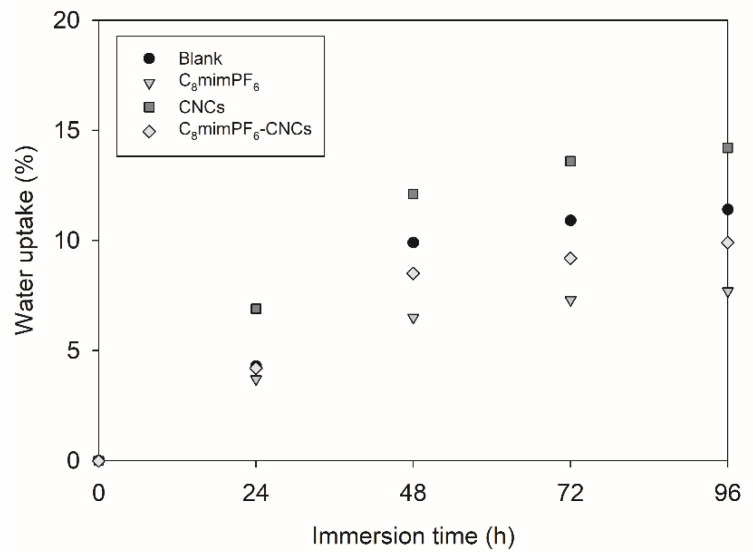
Water uptake of different coated samples immersed for up to 96 h.

In Figure 5, the calculated absorbed water volume fraction at different immersion times is displayed. Waterborne acrylate coatings tend to absorb water due to the existence of a surfactant residue. After 96 h of immersion, the absorbed water volume fraction of the blank sample reached 11.4%. The CNCs sample presented the largest water absorption at 14.2%, which might be due to an enhancement in hydrophilicity caused by the hydrophilic functional groups, such as hydroxyl, carboxyl, and aldehyde groups, inside CNCs [29]. These hydrophilic functional groups favor water retention in the coating. In contrast, the C_8_mimPF_6_ sample exhibited the lowest absorbed water volume fraction of 7.7%, due to existence of a hydrophobic functional group, PF_6_^−^. As a result of this trade-off, the absorbed water volume fraction of the C_8_mimPF_6_-CNCs sample was in between the value of the CNCs and C_8_mimPF_6_ samples. 

The absorbed water volume fraction measures the moisture content retained in the coating. The concept of the electrolyte diffusion coefficient, *D*, is introduced to evaluate how fast the corrosive ions in the water moisture could pass through the coating. The diffusion coefficient, also called the diffusivity, indicates the ability of the corrosive ions to penetrate through the coating. In this study, *D* was calculated using coating capacitance, *CC*, via a simplified Fick’s law of diffusion [3]:

(2) $$\frac{\mathrm{lg}C_{c}-\mathrm{lg}C_{0}}{\mathrm{lg}C_{\infty }-\mathrm{lg}C_{0}}=\frac{2}{L}\sqrt{\frac{D}{\pi }}\sqrt{t}$$

where *C_0_*, *C_c,_* and *C_∞_* are the initial, current, and saturated coating capacitances. *L* is the coating thickness and *D* is the diffusion coefficient. 

**Figure 6:**
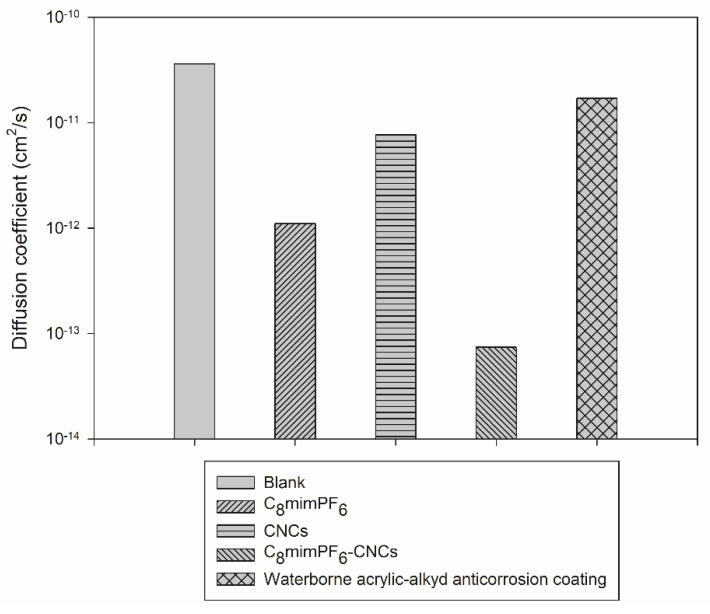
Diffusion coefficients of various coated samples.

From Figure 6, after the introduction of C_8_mimPF_6_ and CNCs, the diffusion coefficient of each sample reduced from $3.6\times 10^{-11}$ to $1.1\times 10^{-12}$ and $7.7\times 10^{-12}$ cm^2^·s^−1^, respectively. The C_8_mimPF_6_-CNCs sample presented the lowest diffusion coefficient of $7.4\times 10^{-14}$ cm^2^·s^−1^, where CNCs enhanced the barrier effect of the PMMA-co-PBA coating film and C_8_mimPF_6_ inhibited the corrosion of the mild steel surface [1,9,10]. In comparison, Ji et al. [30] used the same formula to calculate the diffusion coefficient of corrosive ions in a waterborne acrylic-alkyd anti-corrosion coating and reported that the lowest diffusion coefficient was $1.7\times 10^{-11}$ cm^2^·s^−1^ at 25% alkyd, which is higher than the *D* of the C_8_mimPF_6_ and CNCs samples and much higher than the *D* of the C_8_mimPF_6_-CNCs sample. This result further confirms the synergistic effect of C_8_mimPF_6_ and CNCs on anti-corrosion performance enhancement.

#### 3.1.3. Tafel Polarization Plot

**Figure 7:**
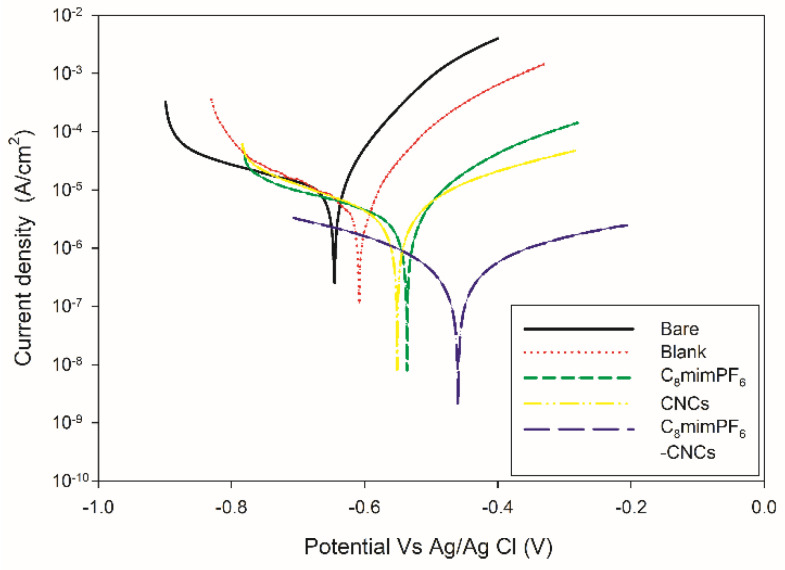
Tafel plots of coated and uncoated samples after 96 h of immersion.

In addition to EIS, Tafel polarization is another electrochemical technique to quantitively evaluate the anti-corrosion performance based on the corrosion potential (*E_corr_*) and the corrosion current density (*i_corr_*). Additionally, *i_corr_* is used to calculate two parameters of anti-corrosion performance: the corrosion inhibition efficiency and the corrosion rate. Figure 7 presents the Tafel plots of coated and uncoated samples after 96 h of immersion. The parameters and calculated variables of these Tafel plots are given in Table 1. Besides the corrosion potential (*E_corr_*) and corrosion current density (*i_corr_*), anodic and cathodic Tafel slopes *b_a_* and *b_c_* were also derived from the Tafel extrapolation of the plot in Figure 7. For bare mild steel, *E_corr_* was −646 mV, and with the coating of neat PMMA-co-PBA, *E_corr_* was −605 mV. After incorporating C_8_mimPF_6_ and CNCs, the *E_corr_* for the C_8_mimPF_6_ and CNCs samples shifted to −534 and −550 mV, respectively. The least negative *E_corr_* was obtained at −467 mV for the C_8_mimPF_6_-CNCs sample. The less negative values of *E_corr_* indicated the enhancement in the anti-corrosion performance [31]. Therefore, *E_corr_* results proved the optimal anticorrosion performance was in the C_8_mimPF_6_-CNCs sample.

As for the corrosion current density (*i_corr_*), instead of a direct comparison, it is used to calculate the corrosion inhibition efficiency (*IE%*) and corrosion rate (*V_corr_*) using Equations (3) and (4), respectively [3]:

(3) $$IE\%=\frac{i_{0}-i}{i_{0}}\times 100\%$$

where *i_0_* and *i* are corrosion current densities of uncoated and coated samples, respectively, after 96 h of immersion at room temperature. 

The *IE%*s were 51% (blank), 70% (C_8_mimPF_6_), 67% (CNCs), and 94% (C_8_mimPF_6_-CNCs) which proved the improvement in anti-corrosion performance under the synergistic effect of C_8_mimPF_6_ and CNCs. Hamidon and Hussin [32] studied the synergistic anti-corrosion impact of a hybrid silane/silicate sol-gel and caffeine in a 3.5 wt% NaCl solution. They stated that the highest inhibition efficiency was 89% at 100 ppM caffeine. Compared with this, our product was better at inhibiting the corrosion of mild steel in 3.5 wt% NaCl. Therefore, the combination of C_8_mimPF_6_ and CNCs would be a competitive method to use in the anti-corrosion of mild steel in 3.5 wt% NaCl solutions. In addition, our C_8_mimPF_6_-CNCs sample was advantageous for the same corrosion inhibition during the immersion period. Murmu et al. [33] used p-phenylenediamine to cure double Schiff base epoxy (DSBE) and applied it to coating of mild steel in a 3.5 wt% NaCl solution. The highest imbibition efficiency was 94% after 24 h of immersion, similar to the *IE%* of our products after 96 h of immersion. 

The corrosion rate (*V_corr_*, mm per year) was also calculated from *i_corr_* using Equation (4), and the results are shown in Table 1 [34].

(4) $$V_{corr}=\frac{i_{corr}M}{DV}\times 3270$$

where *i_corr_* is the corrosion current density (A/cm^2^), *M* is molecular weight (56 g mol^−1^ for mild steel), *V* is the valency (two for the oxidation of mild steel), 3270 (mm·g·A^−1^·cm^−1^·year^−1^) is a constant for unit conversion [35], and *D* is the density (7.85 g cm^−3^ for mild steel). 

The corrosion rate of these samples was consistent with the results of corrosion inhibition efficiency. The results showed that *V_corr_* decreased from 109 µm per year (bare) to 54 µm per year (blank). With the addition of C_8_mimPF_6_ and CNCs, it further decreased to 34 (C_8_mimPF_6_) and 36 (CNCs) µm per year. The synergistic effect of C_8_mimPF_6_-CNCs was further proven with the lowest *V_corr_* of 6 µmm per year, which was 1/18 of the *V_corr_* of the blank sample. In comparison, Cai et al. [36] introduced polyaniline (PANI) and reduced graphene oxide (RGO) as anti-corrosive fillers into a waterborne polyurethane coating and found that the lowest *V_corr_* was achieved at 0.75 wt% RGO/PANI, which was about 1/6 of that of the neat waterborne polyurethane (blank sample). 

#### 3.1.4. Morphology after Immersion Tests

**Figure 8:**
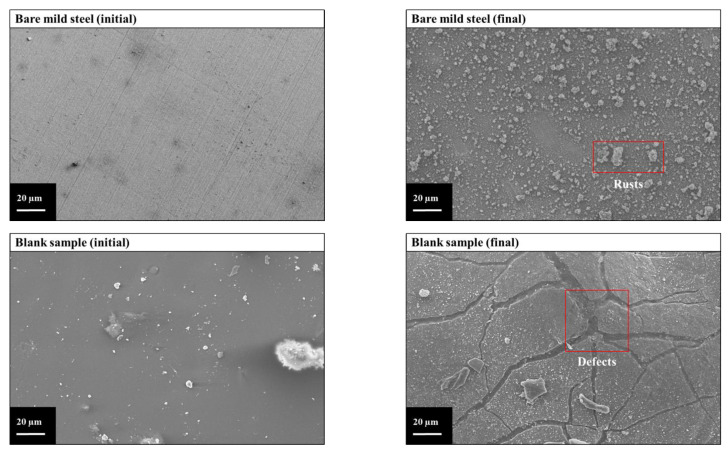

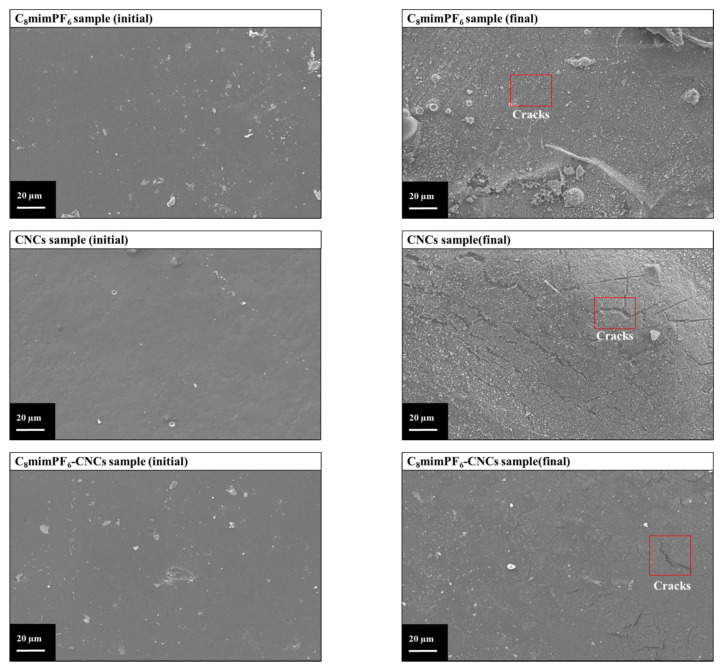
Surface morphology of coated and uncoated samples before and after 96 h of immersion in 3.5 wt% NaCl solution (500 times magnification).

The optimal anti-corrosion performance of the C_8_mimPF_6_-CNCs was further investigated using SEM. The surface morphology with a 500 times magnification for samples before and after immersion was observed and is displayed in Figure 8. Before the immersion test, all coated samples presented a smooth surface with a few residues of copolymer particles on the surface. After the immersion test, rust covered the surface of bare mild steel, which indicated its poor anti-corrosion performance. With the coating of neat PMMA-co-PBA, the surface exhibited some defects instead of the rust observed in the blank sample, which was a sign of anti-corrosion enhancement. After incorporating C_8_mimPF_6_ or CNCs, defects were replaced with a small number of cracks, indicating a further improvement in anti-corrosion properties. With the synergistic effect of C_8_mimPF_6_ and CNCs, the C_8_mimPF_6_-CNCs sample exhibited optimal anti-corrosion performance with sparse cracks and a smooth surface. 

### 3.2. Mechanism of C_8_mimPF_6_ and CNCs in PMMA-co-PBA Anti-Corrosion Coating 

The results above have proved the synergistic anti-corrosion effects of C_8_mimPF_6_ and CNCs in PMMA-co-PBA coatings. To further explain the mechanism of this synergistic effect, the status of the C_8_mimPF_6_ and CNCs in the coating and the wettability of the coating were observed, and a possible detailed mechanism was illustrated with a schematic drawing. 

#### 3.2.1. Status Identification of C_8_mimPF_6_ and CNCs in PMMA-co-PBA Coating

**Figure 9:**
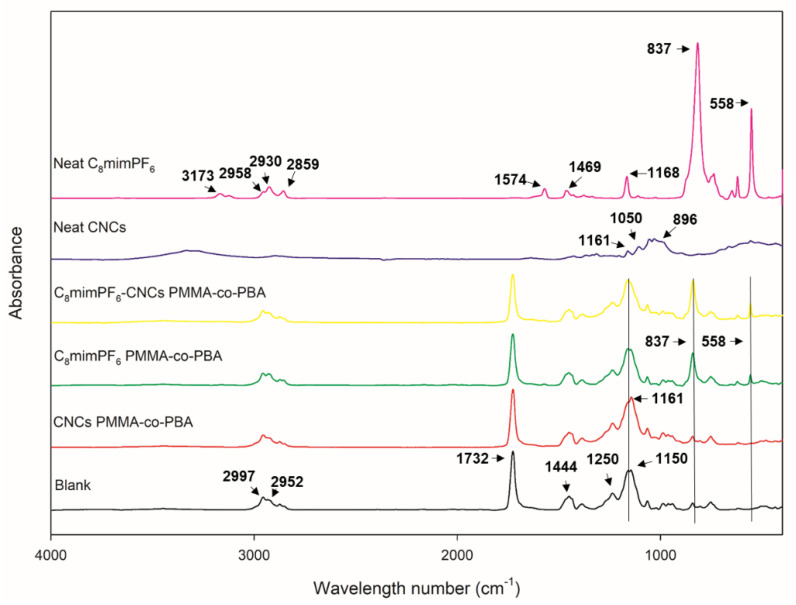
FTIR spectra for various samples.

To identify the status of C_8_mimPF_6_ and CNCs in the PMMA-co-PBA coating, the FTIR spectra of the as-received C_8_mimPF_6_ and CNCs and the as-prepared samples were measured and plotted in Figure 9. For C_8_mimPF_6_, characteristic peaks of C_8_mim^+^ were present at 3173 cm^−1^ (aromatic n(C–H) stretching vibration), 1574 cm^−1^ (imidazolium H–C–C bending), 1469 cm^−1^ (imidazolium H–C–N bending), and 1168 cm^−1^ (imidazolium C2–N1–C5 bending), and the peaks at 837 (P-F stretching vibration) and 558 cm^−1^ (P-F bending vibration) were assigned to PF_6_^−^ [37,38,39]. Meanwhile, the distinct characteristic peaks of CNCs were 1161 cm^−1^ (C-O-C vibration in pyranose ring), 1060 cm^−1^ (C–O–C asymmetric stretching vibration), and 896 cm^−1^ (β-glycosidic linkages of glucose ring) [40,41,42,43]. For neat PMMA-co-PBA, peaks at 2997, 2952, and 1444 cm^−1^ represented the –CH_3_ stretching vibration, the –CH_2_– stretching vibration, and the C–H bending vibration, respectively [44]. In addition, the carboxyl group’s C–O–C stretching vibration induced peaks at 1732, 1250, and 1150 cm^−1^ [44,45]. In the C_8_mimPF_6_ copolymer sample, peaks at 837 cm^−1^ and 558 cm^−1^ were observed, which proved the existence of C_8_mimPF_6_. Moreover, the peak at 1060 cm^−1^ demonstrated the existence of cellulose C–O–C functional groups in the CNCs copolymer sample; the peak might be overlapped by the peak of the carboxyl stretching vibration in the copolymer at 1150 cm^−1^. In the C_8_mimPF_6_-CNCs copolymer sample, these peaks mentioned above were all observed, and confirmed the co-existence of C_8_mimPF_6_ and CNCs. As for the interaction between C_8_mimPF_6_ and CNCs, it has been reported that cellulose can be dissolved in some kinds of ionic liquids [46]. However, the solubility of lignocellulose in imidazolium ionic liquid with PF_6_^−^ anions was extremely low (0.1%) [47]. Therefore, CNCs were not dissolved in C_8_mimPF_6_. Moreover, C_8_mimPF_6_ and CNCs do not contain vinyl or styrene groups. No other new peaks aside from the C_8_mimPF_6_ and CNCs peaks were observed in the spectrum. Thus, it can be concluded that C_8_mimPF_6_ and CNCs did not attend the reaction. Due to the stabilizing properties of CNCs reported in the previous literature [48], CNCs might form strong physical interactions with PMMA-co-PBA. It is speculated that C_8_mimPF_6_ was encapsulated in a PMMA-co-PBA droplet, and CNCs behaved as a co-stabilizer adsorbed on the droplet surface.

#### 3.2.2. Wettability of Copolymer Coating

**Figure 10:**
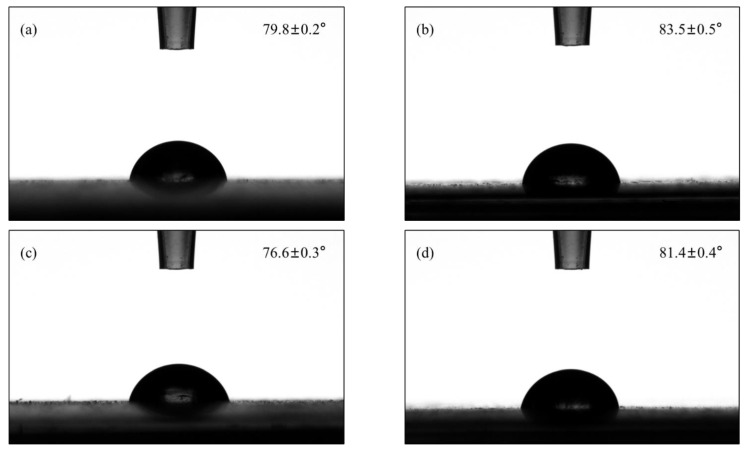
The water contact angle for various coated samples (**a**) blank sample, (**b**) C_8_mimPF_6_, (**c**) CNCs, (**d**) C_8_mimPF_6_-CNCs.

The wettability of the copolymer coating is another concern, because the hydrophobicity of the coating may influence the anti-corrosion performance [49]. Surface roughness and surface chemistry are the two main factors affecting the wettability [50,51]. Based on the results of SEM, the surface morphology of each sample was similar, so the influence of surface roughness on the contact angle was negligible. Therefore, the variation in contact angle obtained for each sample could be ascribed to the surface chemistry. As shown in Figure 10, the water contact angle of the blank sample (neat PMMA-co-PBA) was approximately 79.8°. Due to the typical hydrophobic anion PF_6_^−^, the introduction of C_8_mimPF_6_ improved the hydrophobicity up to a water contact angle of 83.5°. In contrast, the hydrophobicity of the copolymer coating weakened, and the water contact angle decreased to 76.6° after adding CNCs, influenced by the abundance of hydrophilic functional groups such as hydroxyl, carboxyl, and aldehyde groups [26]. As for the C_8_mimPF_6_-CNCs sample, the water contact angle was about 81.4°, located between the values of C_8_mimPF_6_ and CNCs samples. These were consistent with the results of water absorption in Section 3.2.2. The incorporation of C_8_mimPF_6_ and CNCs influenced the wettability of the coating and further affected the water absorption. 

#### 3.2.3. Synergistic Effect of C_8_mimPF_6_ and CNCs in PMMA-co-PBA Anti-Corrosion Coatings 

**Figure 11:**
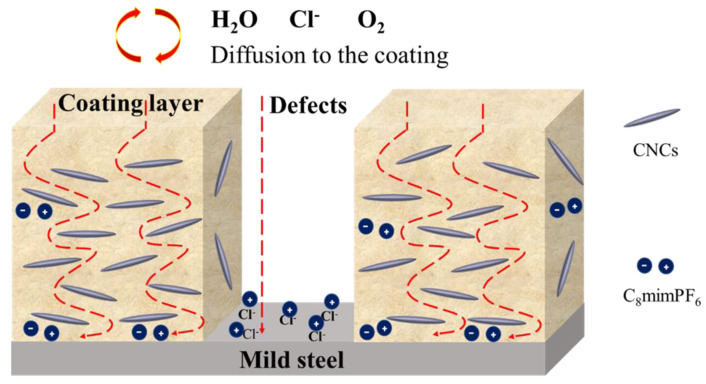
Schematic representation of corrosion protection for mild steel with the C_8_mimPF_6_-CNCs sample.

With all the results listed above, the synergistic anti-corrosion mechanism of C_8_mimPF_6_ and CNCs could be summarized. As shown in Figure 11, at the initial stage of immersion, oxygen, moisture, and other corrosive ions such as Cl^−^ diffuse through the micropores and defects of the copolymer coating to the coating interface and mild steel. Without C_8_mimPF_6_ and CNCs, oxidation and reduction occurred at the interface listed below [52].

Fe → Fe^2+^ + 2e^−^ (5)

Fe^2+^ → Fe^3+^ + e^−^ (6)

H_2_O + (1/2)O_2_(g) + 2e^−^ → 2OH^−^ (7)

2Fe^2+^ (aq) + O_2_(g) + 2H_2_O → 2FeOOH + 2H^+^ (8)

Pitting and delamination of coatings could be induced by these reactions, followed by failure of the protective effect. With the introduction of CNCs, a barrier effect enhancement was observed with a higher coating resistance and lower diffusion coefficient, which might be caused by the reinforcing and impermeable properties of CNCs [9,10]. However, the reduction in the water contact angle and larger water absorption volume ratio compared to the blank sample indicates that there was more water moisture absorbed in the coating. Despite the fact that more water moisture was absorbed, the anti-corrosion performance was still enhanced in the CNCs sample. In other words, most of the water moisture in the CNCs sample was contained to the coating without further penetration through the coating layer. This is similar to other nanofillers, such as nano silica, which can retard the diffusion of water moisture and other corrosive ions to the coating/mild steel interface through zigzagging the pathway [53,54]. Nevertheless, even though the barrier effect of the coating was enhanced, some of the water moisture and other corrosive ions could still pass through the coating and be retained at the interface of coating and mild steel, which could still induce corrosion. With further degradation of the coating film, encapsulated C_8_mimPF_6_ was released and C_8_mim^+^ cations replaced Na^+^, adsorbing electrostatically on the surface of the mild steel due to its larger steric hindrance [1,2,55,56,57]. Then, the corrosion rate was further reduced as the corrosion reaction sites on the mild steel were blocked. In summary, CNCs and C_8_mimPF_6_ worked at different coating sites, where CNCs strengthened the barrier effect of the coating and C_8_mimPF_6_ inhibited the corrosion at the interface of the coating and the mild steel. Therefore, the C_8_mimPF_6_-CNCs composite coating exhibited superior protection than other samples in this research with the highest corrosion inhibition efficiency, the lowest diffusion coefficient and corrosion rate, and the least corroded surface, as observed by SEM. 

As for the interaction between CmimPF_6_ and CNCs, no new peaks were observed in FTIR results. Although it has been reported that either a catalyst or a linker is required at a higher temperature such as 170 °C to graft imidazolium ions onto CNCs [13,58]. Therefore, it could be concluded that no chemical interaction existed between C_8_mimPF_6_ and CNCs in our products.

## 4. Conclusions

A waterborne PMMA-co-PBA latex incorporated with C_8_mimPF_6_ and CNCs was successfully prepared through mini emulsion polymerization. FTIR proved the incorporation of C_8_mimPF_6_ and CNCs, and the anti-corrosion performances of the copolymer coatings were investigated on mild steel samples exposed to 3.5 wt% NaCl. The C_8_mimPF_6_-CNCs sample exhibited a superior anti-corrosion performance with the highest coating, charge transfer resistance, and corrosion inhibition efficiency and lowest diffusion coefficient. The proposed synergistic mechanism showed that the CNCs enhanced the barrier effect of the coating while the C_8_mimPF_6_ inhibited corrosion when released. This study reveals a way to combine two environmentally friendly additives with different anti-corrosive mechanisms to achieve a synergistic effect for anti-corrosion.

## Figures and Tables

**Table 1:** Tafel parameters and calculated corrosion variables of the coated and uncoated samples after 96 h of immersion.

Sample	*E_corr_* (mV)	*I_corr_* (µA/cm^2^)	*b_a_* (V·dec^−1^)	*b_c_* (V·dec^−1^)	Inhibition Efficiency	*R_corr_* (µm per Year)
Bare steel	−646	9.4	61	−334		109
Blank	−605	4.6	82	−222	51%	54
C_8_mimPF_6_	−534	2.9	103	−305	70%	34
CNCs	−550	3.1	138	−287	67%	36
C_8_mimPF_6_-CNCs	−467	0.5	173	−112	94%	6

## Data Availability

The data presented in this study are available on request from the corresponding author.
